# The temporal lagged association between meteorological factors and malaria in 30 counties in south-west China: a multilevel distributed lag non-linear analysis

**DOI:** 10.1186/1475-2875-13-57

**Published:** 2014-02-15

**Authors:** Xing Zhao, Fei Chen, Zijian Feng, Xiaosong Li, Xiao-Hua Zhou

**Affiliations:** 1 West China School of Public Health, Sichuan University, No.17 Section 3, South Renmin Road, 610041 Chengdu, China; 2 Department of Biostatistics, School of Public Health, University of Washington, NE Pacific Street, Seattle 98195, USA; 3 Office for Disease Control and Emergency Response, Chinese Centre for Disease Control and Prevention, NE Pacific Street, 102206 Beijing, China; 4 HSR&D Center of Excellence, VA Puget Sound Health Care System, 1100 Olive Way Metro Park West Suite 1400, Seattle 98101, USA

## Abstract

**Background:**

The association between malaria and meteorological factors is complex due to the lagged and non-linear pattern. Without fully considering these characteristics, existing studies usually concluded inconsistent findings. Investigating the lagged correlation pattern between malaria and climatic variables may improve the understanding of the association and generate possible better prediction models. This is especially beneficial to the south-west China, which is a high-incidence area in China.

**Methods:**

Thirty counties in south-west China were selected, and corresponding weekly malaria cases and four weekly meteorological variables were collected from 2004 to 2009. The Multilevel Distributed Lag Non-linear Model (MDLNM) was used to study the temporal lagged correlation between weekly malaria and weekly meteorological factors. The counties were divided into two groups, hot and cold weathers, in order to compare the difference under different climatic conditions and improve reliability and generalizability within similar climatic conditions.

**Results:**

Rainfall was associated with malaria cases in both hot and cold weather counties with a lagged correlation, and the lag range was relatively longer than those of other meteorological factors. Besides, the lag range was longer in hot weather counties compared to cold weather counties. Relative humidity was correlated with malaria cases at early and late lags in hot weather counties.

Minimum temperature had a longer lag range and larger correlation coefficients for hot weather counties compared to cold weather counties. Maximum temperature was only associated with malaria cases at early lags.

**Conclusion:**

Using weekly malaria cases and meteorological information, this work studied the temporal lagged association pattern between malaria cases and meteorological information in south-west China. The results suggest that different meteorological factors show distinct patterns and magnitudes for the lagged correlation, and the patterns will depend on the climatic condition. Existing inconsistent findings for climatic factors’ lags could be due to either the invalid assumption of a single fixed lag or the distinct temperature conditions from different study sites. The lag pattern for meteorological factors should be considered in the development of malaria early warning system.

## Background

Malaria is an important cause of death and illness in children and adults in tropical countries. Globally, the World Health Organization estimates that in 2010, 219 million clinical cases of malaria occurred, and 660,000 people died of malaria [1]. Despite significant reductions in the overall burden of malaria in the 20th century, malaria remains a significant public health issue in China, especially in the southern part of the mainland. Particularly, Yunnan Province used to be the highest endemic province [2]. For south-west China, the majority of previous studies focused on spatiotemporal pattern for mortality or morbidity [3-5], or pathogenic classifications of reported cases [6].

Malaria is transmitted by female mosquitoes of the genus Anopheles, and the transmission and prevalence of malaria are influenced by many factors, in which meteorological factors are considered to play a crucial role. However, researchers still have a poor understanding of the mechanistic link between climate and malaria risk [7-10]. Many studies were conducted to explore the link with inconsistent findings reported, and the nature and extent of the link remains highly controversial [11-14]. See [7] for a recent review of existing studies supporting and rebutting the role of climatic change as a driving force for highland invasion by malaria. Some existing studies in China made contradictory conclusions [15]. For example, while [16-18] found that rainfall was closely related to malaria incidence, [19-21] failed to identify such an association. Similar inconsistent results were also reported in sub-Saharan Africa [22].

Biologically speaking, climate is fundamentally associated to the malaria incidence through its effects on both the mosquito vector and the development of the malaria parasite inside the mosquito vector. Two aspects of the meteorological effects require special attention, the lag and non-linear characteristics. On the one hand, most time series studies have provided evidence of an association between meteorological variables and malaria, typically at a single lag of 0, 1 or 2 months [23-27]. However, the single fixed lag assumption was not plausible for describing population level associations. From the perspective of biological mechanism, there are several periods need to be considered for the lag effect, such as the time for mosquito to develop, the development period of parasites within the mosquito, and the incubation period of parasites within human body. Every stage shall show a variation in terms of the time lag, resulting in a smoothly varied lag distribution at population level between climatic factors and malaria cases. On the other hand, the non-linear effect was recognized in temperature, and substantial existing studies validated the nonlinear correlation between temperature and malaria in terms of laboratory and epidemiological studies [13,18,28-30]. Similar potential non-linear correlations were also proposed to rainfall [30-32].

The association between malaria and meteorological factors is complex due to the above two characteristics. Existing inconsistent findings may be due to two reasons. On the one hand, regional variations makes distinct regions have different association patterns. On the other hand, invalid statistical assumptions result either from the misspecification of the single fixed lag or from the invalid assumption of the linear relationship.

At present, there are few studies regarding the pattern of delayed effect for meteorological factors after accounting for the nonlinearity. Besides, the comprehensive lag pattern for meteorological factors have not been examined in China. [33] investigated the lag pattern for rainfall in Anhui Province in China using monthly data. However, it is not satisfying, since monthly data was relatively coarse for the lag pattern, and other crucial meteorological variables were not included in the analysis.

The purpose of this work is to explore the lag association between meteorological variables and malaria in south-west China. Specifically, a Multilevel Distributed Lag Non-linear Model (MDLNM) was used to study the temporal lagged correlation between weekly malaria cases and weekly meteorological factors using the data from 2004 to 2009 in 30 counties in south-east China. Using these more reasonable models a better understanding can be obtained for the association between climatic variables and malaria transmission, testing the biological hypothesis in terms of epidemiological level. Also, the result may have the potential to improve forecasting of changes in malaria incidence, which would shed light to public health authorities on how to effectively distribute resources for malaria control programmes.

## Methods

### Study sites

**Figure 1:**
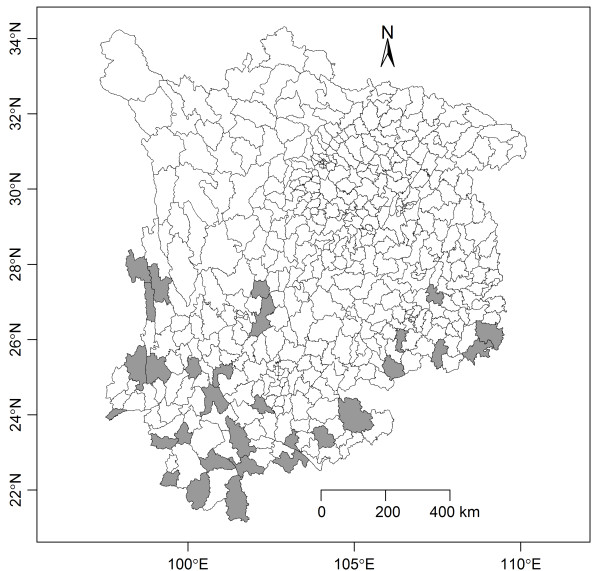
**The map of the 483 counties in south-west China and the selected 30 counties.** The gray-colored counties are the 30 top incidence counties with both malaria and meteorological data.

South-west China (21°14′to 34°31′N, 97°35′to 110°19′E) consists of four provinces, Sichuan, Chongqing, Yunnan and Guizhou. The area has a population of 189,977,077 (sixth national census in 2010) and encompasses 1,137,570 square kilometres. There are 483 counties (county-level cities and districts). 30 counties were selected as the study sites based on availabilities of malaria and meteorological data. The malaria data covered the 483 counties while only 131 counties had the daily meteorological record; the detailed description of these datasets is in the next section. The set of counties with both malaria and meteorological data were sorted by the average annual incidences, and the top 30 counties were included in the analysis. See Figure 1 for a map of the 483 counties in south-west China and the selected 30 counties.

### Data collection and management

Meteorological data were collected from the publicly available Chinese Meteorological Data Sharing Service System [34]. This system was constructed by Chinese National Meteorological Information Centre. There are 836 meteorological monitoring stations with the daily record in the whole China, 131 in the Southwest. Approximately 3–4 counties (438/131) share one monitoring station to monitor the daily meteorological information, and no counties have two monitoring stations. The monitoring station should suffice to represent the county where it is. This assumption has been made substantially in existing studies, both for malaria [4,16-18,20,27,35] and other mosquito-borne diseases [36]. As mentioned in the last section, those monitoring stations located in the high incidences counties and corresponding counties were used. Four weekly meteorological data from July 2003 to December 2009 were obtained for the 30 selected counties. They are rainfall, mean relative humidity, mean minimum temperature and mean maximum temperature. Rainfall and temperatures variables are in the unit of millimetres (mm) and centigrade (°C) respectively.

Weekly malaria cases in the 30 counties were obtained from 2004 to 2009 from Chinese Centre for Disease Control and Prevention (CCDC). At the county level, it is not unreasonable to assume that malaria heterogeneity is not great, which is a usual assumption from existing studies [5,37,38]. In addition, as the interest is on the effect of climatic variables, the heterogeneity caused by other factors should not influence the result, unless other factors are related to the meteorological variables. The malaria data collection was facilitated by Chinese Information System for Infectious Diseases Control and Prevention (CISIDCP). CISIDCP was established on the basis of individual cases and public health emergencies. A Virtual Private Network (VPN) was constructed, and information of individual cases is directly reported to the national database through the internet. This system covers all health data sources and will report new malaria cases to CCDC within 24 hours [39]. Although malaria cases observed in the 30 counties including *Plasmodium vivax* and *Plasmodium falciparum*, most data did not separate different parasites. Population data for every county from 2004 to 2009 were retrieved from the National Bureau of Statistics of China.

### Stratification by temperature

The 30 counties were divided into 15 hot weather and 15 cold weather counties according to the mean minimum temperature, in order to examine the differences between these two groups. Moreover, this approach will lead to more reliable and precise estimates for a given condition, making more generalizable results within similar climatic conditions.

### Multilevel distributed lag non-linear models

The methodology of DLNMs was originally developed for time series data, and a thorough methodological overview was given in [40-42]. Distributed Lag Non-linear Models (DLNM) represent a modelling framework to describe simultaneously nonlinear and delayed dependencies. To get the basic idea on DLNM, in this section, the model included just one meteorological factor, the rainfall. The extension to the other factors is straightforward and shall be presented in the next section. The expected number of cases *E*(*Y*_*it*_) in week t in county *i* were modelled by the Poisson regression,

(1) $$\begin{array}{c} \log\left(E\left(Y_{\mathrm{it}}\right)\right)=\log\left(d_{\mathrm{it}}\right)+\beta_{i0}\\ \;+\overset{L_{\max r}}{\underset{l=L_{\min r}}{\sum }}f\left(x_{i\left(t-l\right),r},\beta_{\mathrm{rl}}\right) \end{array}$$

Here, *d*_*it*_ is the population in county *i* in week *t*; *β*_*i*0_ is the intercept effect for county *i*; *L*_min *r*_ and *L*_max *r*_ represent the minimum and maximum range for the lag effect; and *x*_*i*(*t* - *l*),*r*_ is the rainfall in county *i* in week (*t* - *l*).

Model (1) involves two basis functions for the non-linear and lag effects, respectively. One function is *f*(*x*_*i*(*t* - *l*),*r*_, *β*_*rl*_), which is the non-linear effect of the rainfall *l* weeks before. Many functional forms can be chosen for *f*(*x*_*i*(*t* - *l*),*r*_, *β*_*rl*_), such as polynomial function. The other function is to constrain the parameter *β*_*rl*_. Since there is substantial correlation between rainfall on weeks close together, the above regression will have a high degree of collinearity, which will result in unstable estimates of the individual *β*_*rl*_^'^*s*.To gain more efficiency and more insight into the distributed effect of rainfall over time, it is useful to constrain the *β*_*rl*_^'^*s*. If this is done flexibly, substantial gains in reducing the noise of the unconstrained distributed lag model can be obtained, with minimal bias [43].

The next section concentrates on the choices of two basis functions and the range of the lag effect, *L*_min *r*_ and *L*_max *r*_.

### Lag range specification and other implement issues

The ranges of lag effects for the four meteorological variables were chosen according to [44], which gave an extensive overview of the lag range based on laboratory findings. 3–10 weeks were considered for temperatures. For the rainfall, instead of 4–12 weeks in [44], 4–15 weeks were specified to account for the possibility of longer range, which were reported in existing studies [32,45]. Relative humidity adopted the same lag range as rainfall.

The 3rd-order polynomial was used for both the non-linear and lag effects of meteorological variables. This choice was partly due to the flexibility of the 3rd-order polynomial and partly due to the requirement of parsimony.

Correlations within one county would be greater over those between counties due to some unmeasured (or perhaps unmeasurable) county-specific covariates, and therefore *β*_*i*0_ took a multilevel structure random intercept, which was a normal distribution with a mean of *β*_0_ and a variance of $\sigma_{0}^{2}$.

Including all meteorological variables results in the final model

(2) $$\begin{array}{c} \log\left(E\left(Y_{\mathrm{it}}\right)\right)=\log\left(d_{\mathrm{it}}\right)+\beta_{i0}\\ \;+\overset{15}{\underset{l=4}{\sum }}f\left(x_{i\left(t-l\right),r},\beta_{\mathrm{rl}}\right)\\ \;+\overset{15}{\underset{l=4}{\sum }}f\left(x_{i\left(t-l\right),h},\beta_{\mathrm{hl}}\right)\\ \;+\overset{10}{\underset{l=3}{\sum }}f\left(x_{i\left(t-l\right),T_{n}},\beta_{T_{n}l}\right)\\ \;+\overset{10}{\underset{l=3}{\sum }}f\left(x_{i\left(t-l\right),T_{x}},\beta_{T_{x}l}\right) \end{array}$$

$$\beta_{i0}\sim N\left(\beta_{0},\sigma_{0}^{2}\right),$$

where *x*_*it*,*h*_, $x_{\mathrm{it},T_{n}}$ and $x_{\mathrm{it},T_{x}}$ denote the average relative humidity, the average minimum temperature and the average maximum temperature in county *i* in week *t*, respectively. *β*_0_ is the average intercept over all counties, and $\sigma_{0}^{2}$ characterizes the variation of county-specific intercepts around the average intercept.

One consequence of the stratification by temperatures was that the two groups do not have the same range for the meteorological factors, especially for the temperatures. Besides, the lag pattern could be distinct at different meteorological values. For example, the lag pattern for weekly rainfall might differ between 13.1 mm weekly rainfall and 26.1 mm weekly rainfall. To deal with these two issues, we selected three equally-spaced values on the highly overlapped intervals for the two groups of four meteorological variables, to make the two groups comparable and to reveal the pattern of lag effects over different meteorological variables. Zero was used for all four climatic factors as the reference value to report the result.

Lastly, as sensitivity for the choice of constrained lag function, we also investigated different functional forms fit for the lag effect. Particularly, the 4th order polynomial and B-spline were fitted. The results showed no significant change. All the implementations above were accomplished by R. R is a free software programming language and a software environment for statistical computing and graphics [46]. Specifically, we used two add-non packages, dlnm [47] and lme4 [48].

## Results

### Descriptive analysis

**Figure 2:**
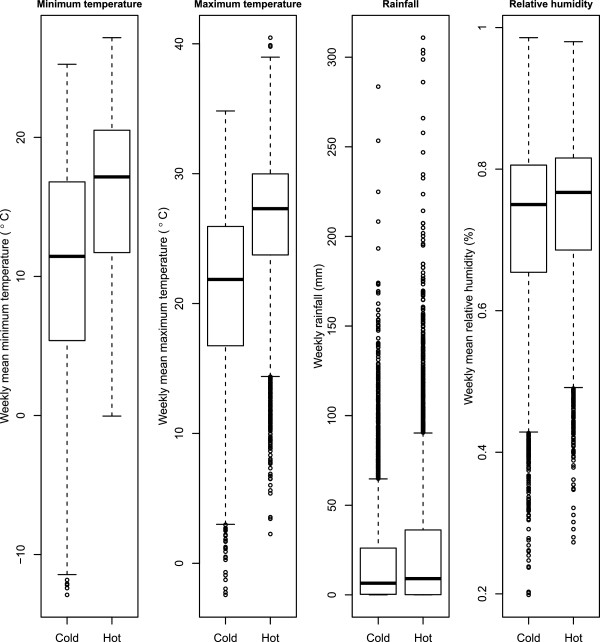
**The box plot comparison of meteorological variables between hot and cold weather counties.** The dark line in the middle of the boxes is the median value; the bottom and top of the boxes indicates the 25th and 75th percentile respectively; whiskers represents 1.5 times the height of the box; and dots with numbers represent value of outlier cases.

A total of 21,944 malaria cases were reported in the selected 30 counties in south-west China from 2004 to 2009. Table 1 presents the descriptive analysis for the 30 counties, while Table 2 shows the Spearman correlation between meteorological variables. Minimum temperature was positively correlated with the remaining climatic factors, with the greatest correlation with maximum temperature (r = 0.782). Maximum temperature showed a weak correlation with rainfall and relative humidity. Finally, rainfall and relative humidity had a relatively strong correlation (r = 0.585). In this part, the result of the test of significance was omitted, as the huge sample size will always lead to a small P value, which is non-informative [49]. Figure 2 demonstrates the comparison of meteorological variables between hot and cold weather counties. While temperatures present a distinctive difference between the two groups, rainfall and relative humidity show similar distributions.

**Table 1:** Characteristics of the 30 study counties

**County**	**Cases**	**Annualized average incidences (/100000)**	**Minimum temperature☆ (**°**C)**	**Maximum temperature☆ (**°**C)**	**Rain☆☆ (mm)**	**Relative humidity☆ (%)**	**Group**
Ruili	3,442	348.204	17.1 (12.4, 21.8)	27.9 (25.3, 30.5)	26.53 (0, 43.03)	73.0 (67, 80)	Hot
Tengchong	9,255	246.049	16.1 (11.2, 21.1)	26.8 (23.3, 30.5)	17.80 (1, 24.7)	65.0 (54, 77)	Hot
Gongshan	300	136.897	2.5 (-3.1, 8.8)	13.4 (8.8, 18.5)	12.29 (2, 17.63)	69.6 (61, 80)	Cold
Fugong	455	80.657	7.5 (1.3, 14)	19.9 (15.8, 24.2)	16.59 (2, 27.85)	67.6 (58, 78)	Cold
Mengla	1,203	79.980	18.1 (14.1, 22.1)	29.4 (27.4, 32)	28.02 (0, 41.83)	80.6 (77, 85)	Hot
Cangyuan	859	65.931	14.9 (9.8, 20.1)	27.2 (24.7, 29.8)	23.63 (0, 37.93)	72.4 (65, 81)	Hot
Menglian	735	55.274	15.4 (10.7, 20.3)	27.6 (25.4, 30)	32.56 (0, 51.6)	75.3 (70, 82)	Hot
Jinping	966	47.375	13.8 (10.1, 18.1)	21.1 (17.4, 25.6)	28.95 (2.25, 40.35)	84.8 (81, 91)	Hot
Longyang	1,976	37.041	11.8 (6.2, 17.6)	23.1 (19.9, 26.4)	17.94 (0.08, 27.23)	73.1 (66, 81)	Cold
Congjiang	688	34.928	15.9 (9.8, 22.6)	24.5 (17.9, 32.1)	22.03 (0.7, 33.8)	78.6 (73, 84)	Hot
Jiangcheng	142	22.283	15.6 (11.7, 19.8)	25.5 (23.1, 28.6)	41.89 (0.38, 69.28)	79.2 (76, 84)	Hot
Menghai	420	21.036	18.4 (14.5, 22.5)	30.2 (27.9, 32.8)	23.17 (0, 38.63)	77.4 (72, 84)	Hot
Weixi	174	18.725	1.7 (-4.9, 9.4)	14.1 (10, 18.5)	11.76 (0, 17.93)	65.9 (58, 74)	Cold
Shuangjiang	113	10.580	13.7 (9, 18.7)	25.0 (22.7, 27.7)	21.22 (0.08, 32.63)	67.6 (59, 77)	Hot
Simao	119	8.132	15.4 (11.5, 19.4)	25.5 (23.2, 28.2)	27.18 (0, 43.8)	75.9 (71, 83)	Hot
Mojiang	173	7.565	19.5 (15.1, 24)	31.1 (27.5, 35.2)	15.40 (0, 21.4)	66.6 (59, 75)	Hot
Jingdong	166	7.382	14.3 (9.3, 19.8)	26.7 (23.7, 30.2)	22.21 (0.38, 31.23)	74.7 (70, 82)	Hot
Dechang	86	7.335	12.9 (8.1, 17.9)	24.3 (19.9, 29.1)	18.39 (0, 28.1)	59.3 (50, 71)	Cold
Gejiu	156	5.535	16.1 (12.3, 20.1)	24.8 (21.8, 28.6)	15.90 (0, 21.18)	68.3 (63, 75)	Hot
Dushan	102	4.984	12.7 (6.7, 19.3)	20.0 (14.4, 26.7)	23.94 (1.5, 32.68)	79.4 (73, 88)	Cold
Changshun	53	3.598	13.0 (7.3, 19.1)	21.2 (15.3, 28.1)	22.30 (1.48, 29.1)	77.5 (72, 84)	Cold
Liping	75	2.522	13.1 (6.2, 20.5)	21.0 (14.2, 28.8)	23.68 (1.9, 33.33)	81.3 (74, 90)	Cold
Wenshan	64	2.325	12.8 (8.8, 17.5)	22.4 (19.5, 26.7)	17.64 (0.5, 26.03)	78.3 (74, 85)	Cold
Wangmo	29	1.609	16.5 (11.5, 22)	25.6 (20.5, 32)	22.43 (0.5, 26.43)	73.2 (67, 80)	Hot
Guangnan	74	1.575	13.6 (8.6, 18.8)	23.5 (19.7, 28.6)	16.98 (0.5, 23.8)	76.8 (72, 84)	Cold
Weishan	28	1.482	11.1 (6.2, 15.9)	21.6 (18.3, 25.3)	20.37 (0, 33.28)	66.2 (55, 78)	Cold
Nanhua	19	1.318	12.0 (7, 16.9)	22.4 (19.1, 26)	15.66 (0, 24.08)	68.2 (59, 80)	Cold
Weng’an	32	1.263	12.9 (6.6, 19.6)	20.2 (13, 28.2)	19.65 (2.6, 27.85)	77.1 (71, 85)	Cold
Eshan	11	1.156	11.8 (7.3, 16.7)	22.6 (19.6, 26.3)	16.23 (0, 23.63)	72.6 (67, 81)	Cold
Huili	29	1.089	10.6 (4.9, 16.7)	22.6 (18.8, 26.6)	21.53 (0, 27.95)	68.0 (60, 77)	Cold

☆: weekly mean, and the two values in the parenthesis are 25% and 75% percentiles, respectively.

☆☆: weekly total, and the two values in the parenthesis are 25% and 75% percentiles, respectively.

**Table 2:** Spearman correlation coefficients between meteorological variables

	**Minimum temperature**	**Maximum temperature**	**Rainfall**	**Relative humidity**
Minimum temperature	1	0.782	0.304	0.278
Maximum temperature	0.782	1	-0.039	-0.132
Rainfall	0.304	-0.039	1	0.585
Relative humidity	0.278	-0.132	0.585	1

Based on Figure 2, three common values for each meteorological variable were selected, to make the hot and cold weather groups comparable. Take the minimum temperature for example, 11.72°C was the 25% percentile for the weekly mean minimum temperature in hot weather counties, and 16.8°C was the 75% percentile for the weekly mean minimum temperature in cold weather counties. These two values shall be used and their mean value (14.26°C) to report the lag pattern in the next section, as both groups covered these values. Similar manipulations were also implemented for the other three meteorological variables.

### Multilevel distributed lag non-linear models

**Figure 3:**
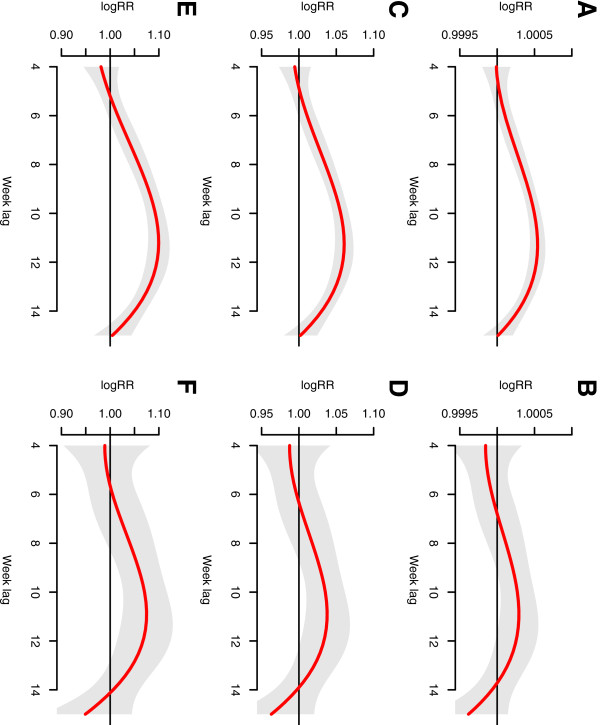
**The estimates of distributed lag between rainfall and malaria cases.** The red line is the estimated distributed lag, with shaded bands indicating its 95% confidence interval. **A** &**B** show the scenario for 0.1 mm weekly rainfall; **C** &**D** show the scenario for 13.1 mm weekly rainfall, and **E** &**F** show the scenario for 26.1 mm weekly rainfall. **A**, **C** and **E** are in the hot weather counties, while **B**, **D**, **F** are in cold weather counties.

Figure 3 shows the estimates of distributed lag between rainfall and malaria cases. First, the distributed lag curves have the same overall trend, an inverse-U shape, with the estimated relative risk increasing at the first half and decreasing at the second half. For the hot weather counties, the correlation gets significant at approximately the 7th week, peaking during the 11-12th weeks and ending with a non-significant correlation at the last week. Besides, the range of significant correlations in cold weather counties is pronounced shorter than that of hot weather counties. Unlike the hot weather counties, the lag range increases with the increase of rainfall in cold weather counties. Furthermore, at each rainfall value, the hot weather counties present higher relative risks verses those of cold weather counties. In addition, in both hot and cold weather counties the relative risk increases with the increase of rainfall, and the magnitude of the increasing trend is greater at the lower rainfall, almost 100 times from 0.1 mm to 13.1 mm, while increasing little from 13.1 mm to 26.1 mm.

**Figure 4:**
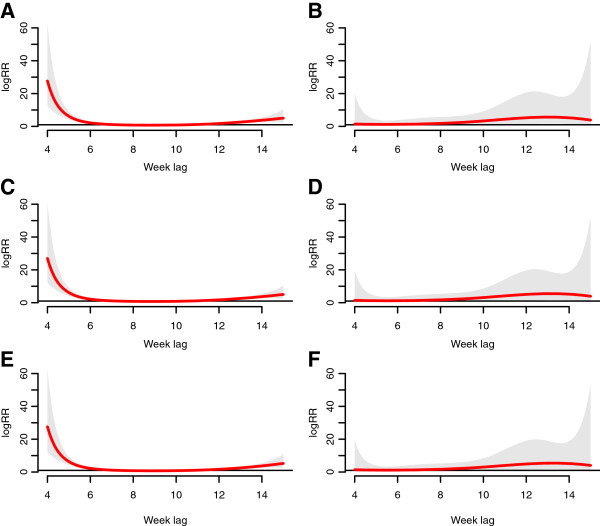
**The estimates of distributed lag between relative humidity and malaria cases.** The red line is the estimated distributed lag, with shaded bands indicating its 95% confidence interval. **A** &**B** show the scenario for 68.57% weekly mean relative humidity; **C** &**D** show the scenario for 74.57% weekly mean relative humidity, and **E** &**F** show the scenario for 80.57% weekly mean relative humidity. **A**, **C** and **E** are in the hot weather counties, while **B**, **D**, **F** are in cold weather counties.

Figure 4 gives estimates of distributed lag relationship between relative humidity and malaria cases. In hot weather counties, there is a significant positive decreasing correlation during the 4-5th weeks, while in the middle the correlation becomes non-significant, and gets significant during the 13-15th weeks. By contrast, the association of relative humidity in cold weather counties is almost not significant over the whole range.

**Figure 5:**
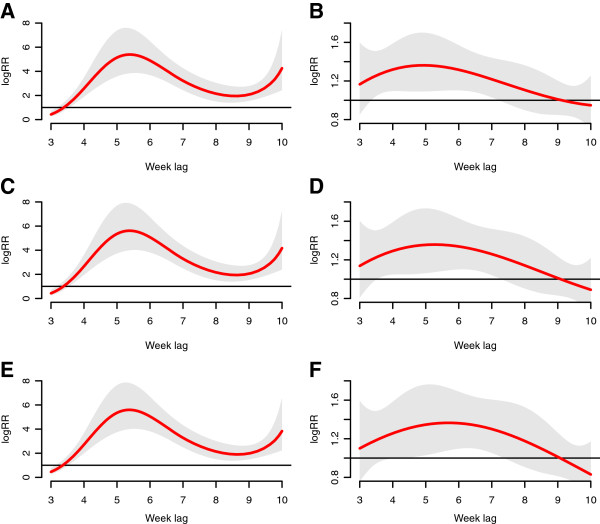
**The estimates of distributed lag between minimum temperature and malaria cases.** The red line is the estimated distributed lag, with shaded bands indicating its 95% confidence interval. **A** &**B** show the scenario for 11.72°C weekly mean minimum temperature; **C** &**D** show the scenario for 14.26°C weekly mean minimum temperature, and **E** &**F** show the scenario for 16.8°C weekly mean minimum temperature. **A**, **C** and **E** are in the hot weather counties, while **B**, **D**, **F** are in cold weather counties.

Figure 5 demonstrates estimates of relationship between minimum temperature and malaria cases. In the hot weather counties, minimum temperature shows a constantly significant association with malaria, usually starting from the 4th week. In contrast, the cold weather counties show a limited range for statistically significant association, usually ending at the 7th week. Besides, at the same minimum temperature value, the correlation in hot weather counties is a greater compared to that of cold weather counties. The 3rd week in hot weather counties shows a statistically negative correlation, but its 95% confidence interval is close to 1.

**Figure 6:**
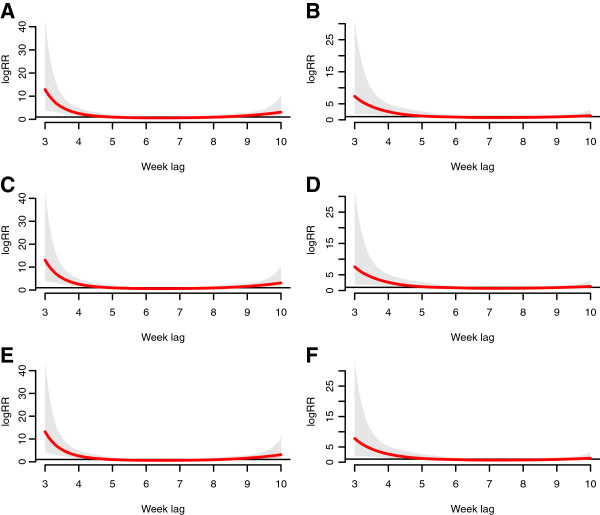
**The estimates of distributed lag between maximum temperature and malaria cases.** The red line is the estimated distributed lag, with shaded bands indicating its 95% confidence interval. **A** &**B** show the scenario for 23.74°C weekly mean maximum temperature; **C** &**D** show the scenario for 24.84°C weekly mean maximum temperature, and **E** &**F** show the scenario for 25.93°C weekly mean maximum temperature. **A**, **C** and **E** are in the hot weather counties, while **B**, **D**, **F** are in cold weather counties.

Figure 6 demonstrates estimates of distributed lag between maximum temperature and malaria cases. The association is similar for hot and cold weather counties, with a significant association during the 3rd-4th weeks.

In Figures 4, 5, 6, the functional form and magnitude of effect are almost the same among three categories of values for the three climatic variables.

## Discussion

Like all mosquitoes, anophelines go through four stages in their life cycle: egg, larva, pupa, and adult [50]. The first three stages are aquatic and also depend on the temperature. The adult stage is when the female Anopheles mosquito acts as malaria vector [51]. Once adult mosquitoes have emerged, the temperature, humidity, and rains will determine their chances of survival. To transmit malaria successfully, female Anopheles must survive long enough after they have become infected to allow the parasites they harbour to complete their growth cycle [52]. Furthermore, a better climatic environment will also shorten the time required for the parasite development in the mosquito (the extrinsic incubation period) [53]. In summary, the meteorological variables can affect the malaria cases through the effect both on every stage of mosquitoes and the parasite within mosquitoes.

The results find that different meteorological factors have distinct patterns and magnitudes for the lagged correlation. Rainfall is associated with malaria cases in both hot and cold weather counties with a delayed correlation and a relatively long lag range, suggesting that rainfall may create collections of water to promote the whole process for the mosquitoes’ development. Furthermore, the lag is longer in hot weather counties compared to cold weather counties, which is biologically plausible, as temperature must be warm enough to support the developments of mosquito and parasites. Besides, although greater rainfall leads to a higher relative risks for malaria cases in both hot and cold weather counties, the increase is greatest when the rainfall is low, while the increase is weaker when the rainfall is high. The saturation effect may be used to explain this phenomenon, in the sense that when rainfall is sufficient, additional rainfall contributes little to the developments of mosquito and parasites.

While relative humidity is not statistically significant in the cold weather counties, the association is statistically significant at early and late lags in the hot weather counties. This could be explained as follows. When the temperature is not low, the relative humidity primarily contributes to the mature of parasites and early development of mosquitoes, respectively.

Minimum temperature has a longer lag range and larger correlation coefficients for the hot weather counties compared to cold weather counties. This is contradictory with some existing studies [44,54], in which they found that small increases in temperature will have a greater effect on malaria transmission in areas with lower average temperatures. This may result from the large difference regarding prevalence rate between the hot and cold weather counties, which can be inferred from the incidence. Hot weather counties have significantly larger incidences than cold weather counties according to Table 1 and the six years incidences for the two kinds of counties, with 407/10,000 and 66/10,000 for the hot and cold weather counties, respectively. Therefore, more infected persons can lead the mosquitoes to a greater exposure chance, which could compensate the less increase effect from minimum temperatures in warmer counties. Maximum temperature is only statistically associated with malaria cases at early lags, implying that maximum temperature may contribute to the mature of the parasite.

The lag functional form is relatively stable for all four meteorological variables within a climatic condition. On the other hand, in terms of the magnitude, the rainfall presents a variation among different values while the other three climatic factors do not show such variation. This indicates the lag pattern is crucial and greatly determines the variation of the effect. Also, it reflects the non-linearity.

Spearman correlation between meteorological variables shows strong correlation between maximum and minimum temperatures on the one hand, and between rainfall and relative humidity on the other hand. This highlights the importance of including a comprehensive set of climatic variables in the model to avoid invalid association.

Existing studies concluded different lags for meteorological factors, and our work gives two possible reasons. On the one hand, existing studies usually assume a fixed lag, and use statistical methods to select the statistically best lag. This approach omits the variation for the lag time, leading to imprecise estimates for the lag. On the other hand, distinct temperature conditions lead to different lag patterns, and therefore existing conclusions are limited to generalize to similar climatic conditions.

The goal of this study is for scientific understanding, not predicting, but the results may provide suggestions for future predicting model. China is implementing a National Malaria Elimination Programme, and the southern border areas will be the one of most hard issue to elimination the disease, particularly in Yunnan [55]. One measure is to predict the malaria cases and release early warning signal when necessary. The results imply that the traditional moving average or fixed lag methods should be modified to weight different time intervals to take account of the biological mechanism.

The nonlinearity was not extensively examined, since the focus is on the lag pattern. Furthermore, it turned out that the lag pattern did not vary significantly with the change of meteorological variables, indicating that the lag pattern is relatively stable within a climatic condition.

This study still has several limitations. First, as with all observational studies for malaria and meteorological factors, it is likely that some confounders influence the result [56]. 30 counties might have different preventive measures (with different magnitudes) to combat malaria, and they may also have different behaviours, such as the use of nets. Including city-specific random effect could not eliminate the potential bias. Second, the quality and completeness of the data may change over the six year period [57-59]. The change mainly occurs in the time dimension, with best quality in 2009 [59]. Third, the finite and pre-defined lag ranges for meteorological variables were used. The lag lengths were chosen mainly according to [44], which gave both the biological reasoning and the empirical study. Finally, the lag pattern of *P. vivax* and *P. falciparum* malaria could be different. As with some existing studies [5], separate analyses by different parasites were not made owing to a lack of detailed information on *P. vivax* and *P. falciparum* in this study. The difference may come from the incubation period, the time between the initial malaria infection and symptoms. However, the incubation period generally ranges from 9 to 14 days for *P. falciparum* and 12 to 18 days for *P. vivax*[60], and therefore the general lag pattern should not differentiate greatly. Nonetheless, further epidemiological researches are warranted to explore the possible different lag patterns.

## Conclusions

Using weekly malaria cases and meteorological information, this work studied the temporal lagged association pattern between malaria cases and meteorological information over six years (2004–2009) in 30 counties in south-west China. The results can be viewed as supplementary information for the existing inconsistent findings on the lag pattern, especially for China, where no similar study was reported before. Different meteorological factors show distinct patterns and magnitudes for the lagged correlation, and the patterns will depend on the climatic condition. Therefore, existing inconsistent findings for climatic factors’ lags could be due to either the invalid assumption of a single fixed lag or the distinct temperature conditions from different study sites. The lag pattern for meteorological factors should be considered in the development of malaria early warning system, and how to incorporate the lag pattern into predicting model is an open question.

## Competing interests

The authors declare that they have no competing interests.

## Authors’ contributions

XZ performed the statistical analysis and drafted the manuscript. XZ and FC cleared the data. ZJF provided the original data. XSL conceived of the project concept. XHZ gave technical assists. All of the authors have read and approved the final manuscript.
